# Combined laparoscopy and transabdominal endoscopy: Case report of dislodged oesophageal stent retrieval

**DOI:** 10.4103/0972-9941.78356

**Published:** 2011

**Authors:** Rajiv K Chander, Kigongo Samuel, Valerie Katz, Mark Ingram

**Affiliations:** Lincoln Hospital Affiliate of Weill Cornell Medical College and New York Medical College, New York, USA; 1 Department of Surgery, Lincoln Medical and Mental Health Center, Bronx, New York, USA

**Keywords:** Oesophageal, laparoscopy, stent, transabdominal endoscopy

## Abstract

The patient is a 39-year-old male with a five-month history of progressive dysphagia and a 70 lb weight loss. On upper gastrointestinal (GI) endoscopy he was found to have a near-obstructing mass in the lower oesophagus that was proven by biopsy to be oesophageal adenocarcinoma. Stricture caused by the adenocarcinoma mass was stented with a Cook Evolution 12.5 cm / 24 Fr stent, which dislodged subsequently. We report the first case of a dislodged Cook Evolution 12.5 cm / 24 Fr oesophageal stent that was retrieved using combined laparoscopic and transabdominal endoscopy.

## INTRODUCTION

Oesophageal carcinoma is a major surgical challenge, with 50 to 60% having incurable disease at the time of presentation. Palliative therapy to relieve debilitating dysphagia remains one of the primary treatments for these patients.[1] In recent times, stents are one of the major palliative procedures offered to patients with obstructive oesophageal cancer. Currently, stents are pertinent to maintain or regain patency in the hollow organs, vessels and ducts. Alimentary tract stenting has a wide variety of uses, which include, oesophageal malignancies, strictures and gastroduodenal and colonic malignant obstruction, where a traditional surgical approach is not an option, due to patient comorbidities, and palliative measures are the end point to therapy. We report the first case of migration of a Cook Evolution 12.5 cm / 24 Fr oesophageal stent that was retrieved using combined laparoscopic and transabdominal endoscopy.

## CASE REPORT

**Figure 1:**
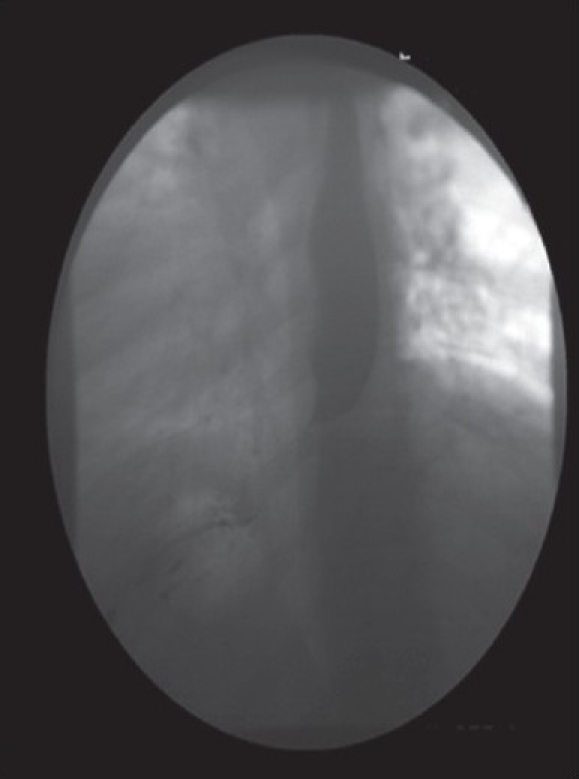
Oesophagram: Barium abruptly stops in distal oesophagus, no mass, trickling of barium into stomach

**Figure 2:**
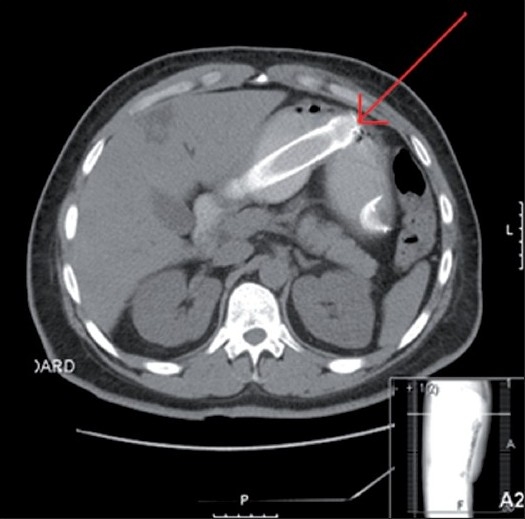
Stent in the distal portion of the gastric body, through the antrum and the pylorus

The patient is a 39-year-old male, with a five-month history of progressive dysphagia and a 70 lb weight loss, with a past medical history significant for diabetes, 20-pack-per-year of smoking and coronary artery disease, with stenting, in 2006. With a high index of suspicion, the patient was worked up with a barium swallow that showed evidence of narrowing of the distal oesophagus [Figure 1].[2] On upper GI endoscopy he was found to have a near-obstructing mass in the lower oesophagus that was proven by biopsy to be oesophageal adenocarcinoma. At a subsequent endoscopy session, the stricture causing mass was stented with Cook Evolution 12.5 cm / 24 Fr stents. Both stents were inserted at the initial session; the first stent was dislodged during placement and could not be retrieved and the second stent was placed at this time. Two days later, the patient presented to the ER with complaints of crampy epigastric pain, nausea and vomiting. A computed axial tomography (CAT) scan of the chest and abdomen showed one stent in the lower oesophagus extending into the fundus of the stomach, and another stent in the distal antrum and the pre-pylorus [Figure 2]. Surgical consult was sought and after considering all risks, benefits and alternatives we decided to perform a laparoscopic gastrotomy for retrieval of the foreign body.

## PROCEDURE

**Figure 3:**
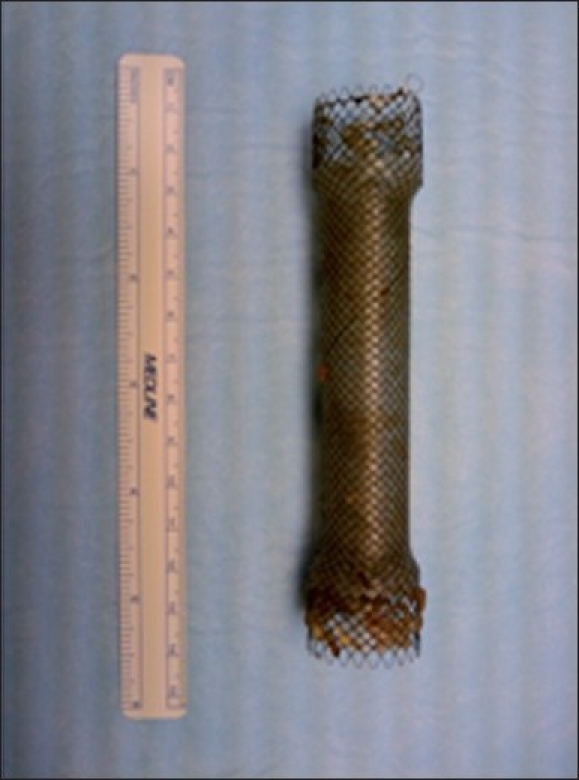
Stent one skewed at GE

Pneumoperitoneum was established with a Veress needle placed at the supraumbilical crease. A 5-mm port was placed to the left of the midline in the epigastria. A 30-degree 5-mm laparoscope was then introduced into the peritoneal cavity. A 15-mm balloon-trocar (1 – 10 / 12 mm × 100 mm Gelport balloon Trocar, Rancho Santa Margarita, Ca. 92668) was placed in the mid-clavicular line below the left costal margin. Another 5-mm port was placed in the left subcostal area. A paediatric gastroscope was introduced via the mouth and upper GI endoscopy was performed to the gastro-oesophageal (GE) junction. The stent was not traversed because we were apprehensive of dislodging it. The stomach was insufflated and a gastrostomy was performed. The stomach was insufflated with air to maximum capacity, to allow for elevation of the anterior gastric wall. The antrum of the stomach was grasped using a Babcock and elevated. A gastrostomy about 1.5 cm in length was made over the antrum of the stomach. The 15-mm balloon-port was placed into the gastrostomy and the balloon was inflated to allow for elevation of the stomach towards the anterior abdominal wall and to seal off the stomach contents. The gastroscope was now withdrawn from the mouth and was introduced through the balloon-port transabdominally, and two stents were noted: one straddling the GE junction and another dislodged stent sitting in the antrum. The dislodged stent was visualised and a snare was introduced through the gastroscope, but the free stent was not able to be snared. Biopsy forceps were introduced at this time through the gastroscope and the disloged stent was withdrawn via the balloon-port [Figure 3]. The gastrostomy was closed using an EndoGIA stapling device via the balloon-port. After closure of the gastrostomy, peritoneal lavage and cytology was obtained (there were no peritoneal or liver metastases). The 15-mm port site was closed using a fascia needle employing a 0-vicryl suture. An nasogastric tube (NG) tube was left in place in the stomach postoperatively for decompression.

## DISCUSSION

In recent years the role of palliative stenting in the management of patients with obstructive or near obstructive oesophageal cancer has expanded. The current stents available for application are self-expanding metal stents and self-expanding plastic stents. The self-expanding plastic stents used in palliation of oesophageal obstruction have a smooth inner surface, for passage of food, and a rough outer mesh to keep the stent in place and potentially prevent migration. The Polyflex stent used frequently in the past may be removed after being in place for a month, which makes it an attractive tool for oesophageal stricture and tracheo-oesophageal fistulas.[3,4] Self-expanding metal stents have been shown to be superior to plastic stents.[5–9] Covered metal stents are coated with silicone or polyurethane, while uncovered metal stents have a bear metal mesh. Uncovered stents often face the disadvantage of tumour ingrowth and subsequent obstruction, whereas, both covered and uncovered can have an overgrowth at their ends. Covered metallic stents avoid ingrowth of tumour through the metal mesh, whereas, 25% of the uncovered stents have the complication of ingrowth.[10] Migration of the deployed stent is one of the complications, whereas, in the earlier models the silicone or polyurethane coating may have prevented integration into the oesophageal wall.[11] The addition of barbs, leaving the proximal and distal ends of the stent uncovered, and the addition of proximal enlarged flange have decreased the risk of migration.

In addition to the standard risks of endoscopy, metal stent placement has additional risks associated with it, such as, several severe, life-threatening complications, including perforation, haemorrhage, and airway compression. Perforation and haemorrhage may be immediate or delayed, while airway compression is an immediate complication, and it is advocated that bronchoscopy and possible tracheal stent placement be performed simultaneously or before oesophageal stent placement for bulky lesions in the upper oesophagus involving or compressing the airways. With metal placement in the oesophagus, perforation and bleeding are the most serious complications of gastroduodenal stent placement, occurring in 0.7 and 0.5% of the patients, respectively.[12] Stent migration (5%) and re-stenosis (18%) are typically late complications, and the majority of these complications can be managed with insertion of an additional stent.[12] One of the most common indications for removal is bowel obstruction. Non-surgical removal of dislodged stents is usually difficult. We present the first case report where such a stent was removed using a minimally invasive technique and avoiding the morbidity associated with a laparotomy and often gastrotomy. Furthermore, this is the first case highlighting the use of transabdominal endoscopy via the balloon-port, which maintains both pneumoperitoneum and visceral distention, while preventing contamination, and allows the use of the gastroscope with its advantages over traditional laparoscopic instrumentation.
